# Current aspects of salivary gland tumors – a systematic review of the literature

**DOI:** 10.3205/iprs000138

**Published:** 2019-08-02

**Authors:** Theresa Marie Galdirs, Matthias Kappler, Waldemar Reich, Alexander W. Eckert

**Affiliations:** 1 Martin Luther University Halle-Wittenberg, University hospital, Department of Oral and Maxillofacial Plastic Surgery, Halle, Germany

**Keywords:** salivary gland tumors, pleomorphic adenoma, adenoid cystic carcinoma, mucoepidermoid carcinoma, acinic cell carcinoma

## Abstract

**Objectives:** This study provides an up-to-date overview of the distribution of salivary gland tumors in relation to sex, land of treatment, localization of the tumor in the mouths, and benign/malignant disease of this type of tumor. We hypothesized that the distribution of patients with salivary gland tumors could vary according to country, gender, age and tumor specificity. In addition there is a comparison of the primary classification of salivary gland tumors from 1981 and the recent classification from 2005.

**Materials** **and methods:** Data from the Medline database PubMed.gov and supplementary sources were used to conduct a systematic literature search. For this purpose, data from different studies were independently collected using a previously designed questionnaire.

**Results:** The first section analyzes the general features of the relevant salivary gland tumors from 141 studies involving a total of 25,826 patients across 30 different countries in terms of gender and the occurrence of benign/malignant salivary gland tumors. These data were summarized and presented.

**Conclusion:** This review offers an insight into the dramatic local differences with regard to salivary gland tumor occurrence as a stepping stone to further classify such data in order to derive effective therapy options, prognosis and widen the general understanding of the subject.

## Zusammenfassung

**Ziel:** Unser Review gibt eine aktuelle Übersicht über das Vorkommen der Speicheldrüsentumore. Näher dargelegt wurde dabei die divergierende Verteilung dieser Tumore bezogen auf Alter, Gender, anatomische Lokalisationen und spezifische geographische Ländereinteilungen. Ebenfalls visualisiert wurden die unterschiedlich auftretenden Entitäten bezogen auf Malignität und Benignität.

Unsere Ausgangshypothese war, dass in verschiedenen Ländern die Verteilung der Tumorentitäten sowie die weiteren Charakteristika der Tumore (z.B. Alter und Gender der betroffenen Patienten, anatomische Lokalisation, Auftrittshäufigkeiten verschiedener Entitäten) variieren.

Zusätzlich gibt unser Review eine Übersicht über die Veränderung der Tumorklassifikationen seit 1981 bis 2005.

**Material und Methoden:** Für die systematische Literaturrecherche wurde die Datenbank PubMed.gov genutzt. Es wurde ein Fragenkatalog erstellt und Studien aus verschiedenen Ländern aufgenommen und analysiert.

**Ergebnisse:** Durch das erste Auswahlverfahren wurden 141 Studien aus über 30 Ländern selektiert. Die somit erfasste Patientenkohorte betrug 25.826. Die Daten aus diesen Studien wurden bezüglich der verschiedenen Länder, Alter, Gender und dem Auftreten unterschiedlicher benigner und maligner Entitäten zusammengefasst und analysiert.

**Schlussfolgerung:** Dieses Review verdeutlicht die starken lokalen Unterschiede im Auftreten der Speicheldrüsentumore. Unsere gesammelten Daten sollten als Grundstein für weitere Forschungen bezogen auf Therapieoptionen, Prognosen und Vorhersagen zu dem Auftreten von Speicheldrüsentumoren genommen werden.

## Introduction

Salivary gland tumors are a rare phenomenon. This heterogeneous group of pathologies encompasses approximately 3–5% of head and neck carcinomas, and only 0.5% of all malignant tumors match these types [1]. The incidence of all salivary gland tumors varies between 0.3 to 4 per 100,000, population, with the highest identified among the Inuit [2], [3]. 

The results of current studies focus on isolated characteristics of the salivary gland tumors (e.g., age and gender, therapy, localization, etc.) as opposed to a holistic view of salivary gland tumors, and therefore, they need to be summarized and visualized to be easily comparable in terms of the epidemiology, therapy, and prognosis. Some different reviews concerning salivary gland tumors have been published in the last two centuries [1], [4], [5], [6], [7], [8], [9], [10], [11], [12], [13], [14], [15], [16], [17], [18] most of which discuss the entities or diagnostic or prognostic aspects and principal therapeutic strategies in detail. However, there is no research article that described an overview of the gender-specific distribution as well as country-specific differences for those tumors. Therefore, the purpose of this actual article is to present an up-to-date overview of the characteristics of salivary gland tumors through a literature review. This overview will detail the epidemiologic, gender, benign/malign and country-specific distributions of this tumor entities. Moreover, this work presents an inventory of the current state with regard to salivary gland tumors in general.

## Material and methods

### Study selection criteria 

The literature search was carried out until January 2018, and includes articles dating back as far as 1981. The databases were searched for relevant studies using the key words “salivary gland tumors”, “pleomorphic adenoma”, “adenoid cystic carcinoma”, “mucoepidermoid carcinoma” and “acinic cell carcinoma”. 

As support, 3 textbooks and 1 medical doctoral thesis were used to complement the article. All the used sources are freely accessible. 

Studies eligible for inclusion in this analysis had to meet the following criteria: recent publications (between 1981 and 2018), statistically evaluable, multiple salivary gland tumor types, various geographic and anatomic locations, therapy analysis, prognosis with a focus on survival (disease-free survival, overall survival, relapse time, among others), case studies, clinico-pathological studies in hospitals, retrospective cohort studies in patients with salivary gland tumors and reviews. 

Two reviewers (TMG and AWE) independently carried out the study selection, data extraction, and quality assessment. The reviewers independently screened all records (titles and abstract) that were identified by the search strategy to select potentially eligible publications. Care has being taken to discard duplicated content, outdated and unrelated studies. Country-based statistics were derived from individual clinic data with a significant amount of patients only. 

### Data extraction

The two reviewers independently extracted data from all eligible studies using a previously designed question catalogue. The following information was compiled: title of the publication, name of the journal, data source, name of the first author, year of publication, country, study design and characteristics of the study findings (age, sex, countries, prognosis), cancer type, cancer site, clinical and pathological tumor stage, type of treatment and survival analysis. Studies of all age groups were included in the analysis.

**Figure 1:**
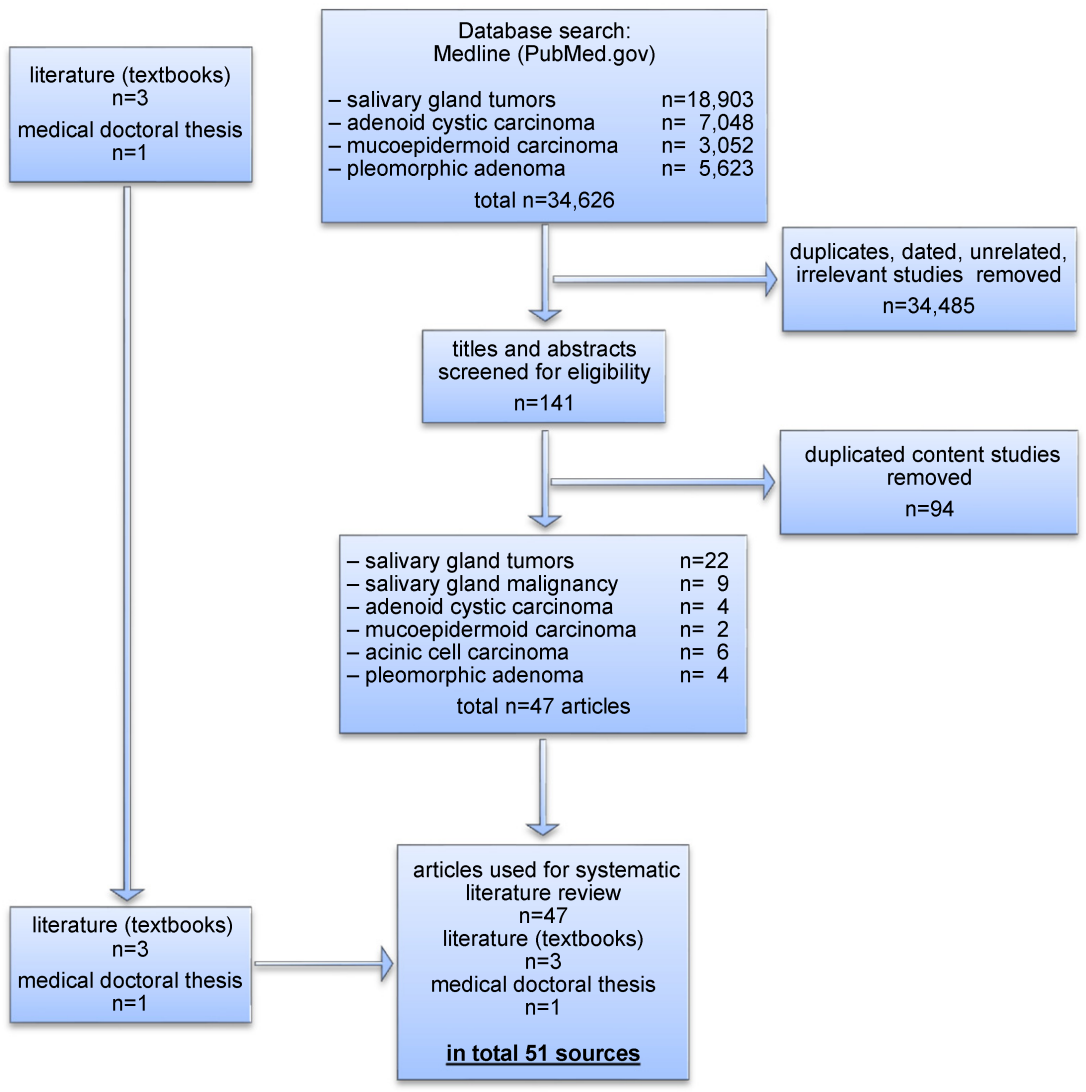
Flowchart of the study selection process

Figure 1 (Fig. 1) shows a flow chart of the identified and included records. The detailed results of characteristics and parameters are presented as follows. 

As shown in Figure 1 (Fig. 1), among thousands of published articles dealing with salivary gland tumors, a total of 51 sources were considered relevant for further investigation. 

## Results

### Epidemiological data 

Patient ages ranged from 2.5 to 92 years [5], [19], even though some studies focused on occurrences in children. The mean age ranged from 41.9 to 43 years [19], [20], correlating with a peak in the fifth decade of life [21]. Benign tumors were more likely in younger patients aged 35.0±17.2 years and malignant tumors in older patients aged 48.8±18.2 [22]. 

Depending on the country of origin, the distributions of salivary gland tumors differed between men and women. In China, the distribution of men/women was 1 : 0.9 [23], and in Nigeria it was 1 : 1.8 [19]. No special data were found for all salivary gland tumors, or specifically for tumors of minor salivary glands in Germany. Table 1 (Tab. 1) shows the detailed gender distribution of different countries.

### Anatomical localization

Between 60 and 84.2% of all tumors were to be found in the parotid glands [6], [20], but only 16% were in the submandibular glands. Tumors of the sublingual gland were rare, but all were malignant according to Luksic et al. [24]. Malignant tumors are generally less common than benign tumors in the large salivary glands [6].

Minor salivary gland tumors were found in 24 to 39.3% of cases [19], [20]. The palate was the most common site of minor salivary gland tumors in 33.3 to 67% of cases [21], [25], followed by the upper lip and buccal mucosa [26]. A larger proportion of benign tumors were found in the palate of females (75.00%) compared with male patients (64.00%) [7].

### Diagnostic approaches

Preoperative diagnostics are mainly based on imaging methods and pathological findings, especially fine-needle aspiration cytology (FNAC) [2].

Ultrasound, magnetic resonance imaging (MRI) and contrast-enhanced computed tomography (CT) are the most commonly used imaging modalities to evaluate salivary gland lesions [27].

Ultrasound remains the basic diagnostic imaging procedure, especially when occurring in parotid glands [2]. As a low cost, non-invasive modality, ultrasound provides excellent localization of the tumor in the gland and enables differentiation from the cystic mass [2].

For lesions of the minor and sublingual salivary glands of the deep parotid lobe or of malignant neoplasia with suspected perineural invasion or bone infiltration, MRI is mandatory to evaluate the tumor extent, local invasion and perineural spread [2].

CT should be an alternative modality when MRI is not available. Certain carcinomas (for example, mucoepidermoid carcinoma, adenoid cystic carcinoma or acinic cell carcinoma) may lack significant contrast enhancement, leading to oversight or underestimation of the lesion [2].

### Tumor type

In total, the evaluation included 9 studies on malignant salivary gland tumors and 22 on benign and malignant. In most studies, pleomorphic adenoma was most likely in benign tumors in approximately 42% of cases [21], [28]. The three prevalent malignant salivary gland tumors are adenoid cystic carcinoma, mucoepidermoid carcinoma and acinic cell carcinoma [29]. The distribution of tumor types broken down by countries is shown in Table 2 (Tab. 2). 

Irregular margins, bony invasions, the presence of metastatic lymph nodes and perineural spread can all be signs of malignancy [30]. Necrosis can also characterize malignancy [31].

### Distribution of malign and benign salivary gland tumors

Examining all salivary gland tumors, the distribution of malignant and benign also differed, as shown in Table 3 (Tab. 3). The distribution also differed for minor and major salivary gland tumors. In Brazil, only 20% of all tumors were malignant [32], whereas this number was much higher in Nigeria, where 71.1% were diagnosed [19]. We found no reliable studies or reviews from Germany because most of the data were from limited patient groups or were not properly itemized.

### Histologic entities of salivary gland tumors

It has been difficult to categorize salivary gland tumors according to their clinical behavior. To retain the kind of tumors, Schwenzer/Grimm and Barnes published a differentiated WHO histological classification of tumors of salivary glands in 1981 [33] and in 2005 [34] (Table 4 (Tab. 4)).

### Therapeutic aspects of salivary gland tumors

Salivary gland carcinomas are tumors with a heterogeneous morphology that require distinctive surgical therapy [2]. Surgical excision is the treatment of choice for resectable tumors [2]. Chemotherapy for salivary gland tumors can be ineffective. Studies of newly targeted therapies have not offered significant benefits [35]. Successes with chemotherapy alone and/or combinations with radiotherapy have been recorded for salivary duct carcinoma and carcinoma ex pleomorphic adenoma [36]. Histologic grade is important for prognosis and therapy. Surgery remains the mainstay of treatment when negative margins can be achieved. Radiation improves locoregional control of tumors with high-risk features [35]. In conclusion, more additive prognostic parameters are of great interest. This individual molecular signaling is further discussed in detail.

## Discussion

With the aforementioned key words and generally available research mechanisms, e.g., PubMed.gov, more than 37,000 publications could be identified. At first glance, this seems to represent a unique treasure trove of data. However, applicable and valuable data were extracted by careful categorization and selection, which is necessary because articles that present a current overview of benign and malignant salivary gland tumors are missing. 

Despite differing countries of origin, our work identified several similarities. For example, benign tumors were more common than malignant ones. The prevalent benign tumor was PA, and the prevalent malignant tumors were ACC and MEC [5], [6].

The majority of tumors in the minor salivary glands and in the sublingual gland were malignant [7], [11].

The significance of the evaluated literature is indicated, but it differs for every country and every tumor entity. In some countries, the studies include thousands of patients, whereas in other countries, the studies include only up to a few hundred patients. The studies are often of one single institute and do not evaluate the whole country. Tumor research also differs. Most research concerns the most prevalent tumors (e.g., PA), but more attention should be focused on malignant ones because a permanent and frequently extended overview about therapy, prognosis and the distribution of different features (e.g., gender, age, proportion of malign and benign) is currently needed for improved diagnosis and treatment in the future. This could also provide insights into the formulation of risk groups to receive recurring preventive examinations. In all reviewed publications, however, none showed a correlation with cultural standings, living circumstances or habits (e.g., smoking, alcoholism). 

To the best of our knowledge, this is the largest research article of salivary gland tumors in terms of gender distribution. Moreover, this work primarily presents a scientific summary of the worldwide distribution of benign and malignant salivary gland tumors, their demographics, the distribution of multiple entities in the form of a mini review, as well as the clinical features (e.g., symptoms, therapy, prognosis) of general salivary gland tumors. To our knowledge, this is the first article to contain all of these characteristics, thus providing a specific view perspective.

By comparing these results with squamous cell carcinoma (SCC) of the oral cavity, for which such questions have been undergoing assessments with great success for almost two decades, the research activity for malignant salivary gland tumors is in its infancy.

This article reveals an apparent lack of research in Germany. A thorough analysis from a maxillofacial surgical standpoint is also missing. A next step will thus be to perform a monocentric study of this topic, particularly because prognostic assertions of benign and malignant forms (PA, ACC, MEC, AciCC) will be of great clinical importance. The University of Halle (Saale) is currently evaluating its diagnosed tumor entities over the last 25 years to provide this missing information. However, the results must be combined with other university findings to generate a holistic view of different tumor entities in Germany.

## Notes

### Acknowledgements

We thank our colleagues from the Department of Oral and Maxillofacial Plastic Surgery for contributing to this study and for their continuous support.

### Competing interests

The authors declare that they have no competing interests.

## Figures and Tables

**Table 1:**
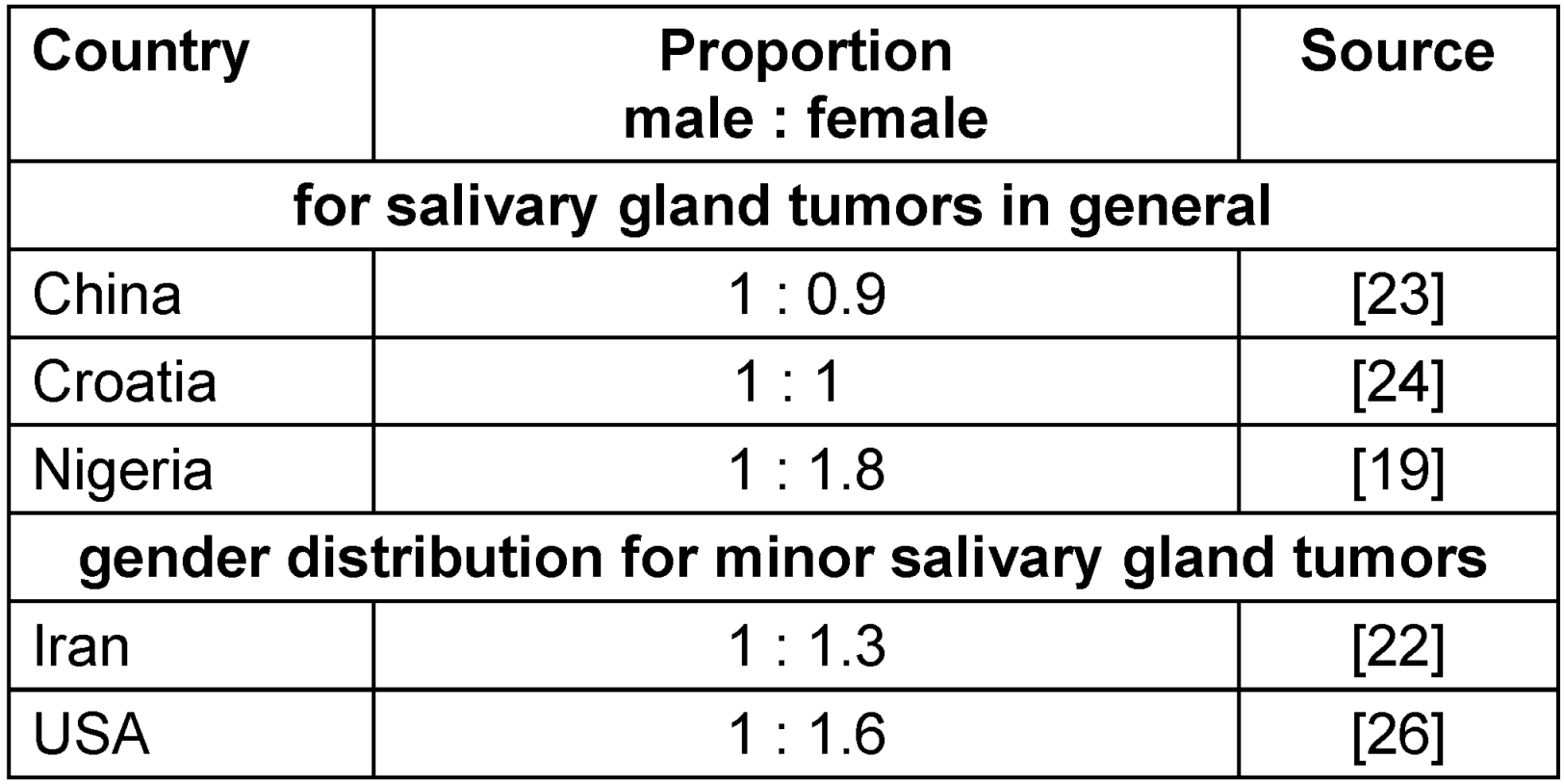
Gender distribution of different countries in detail

**Table 2:**
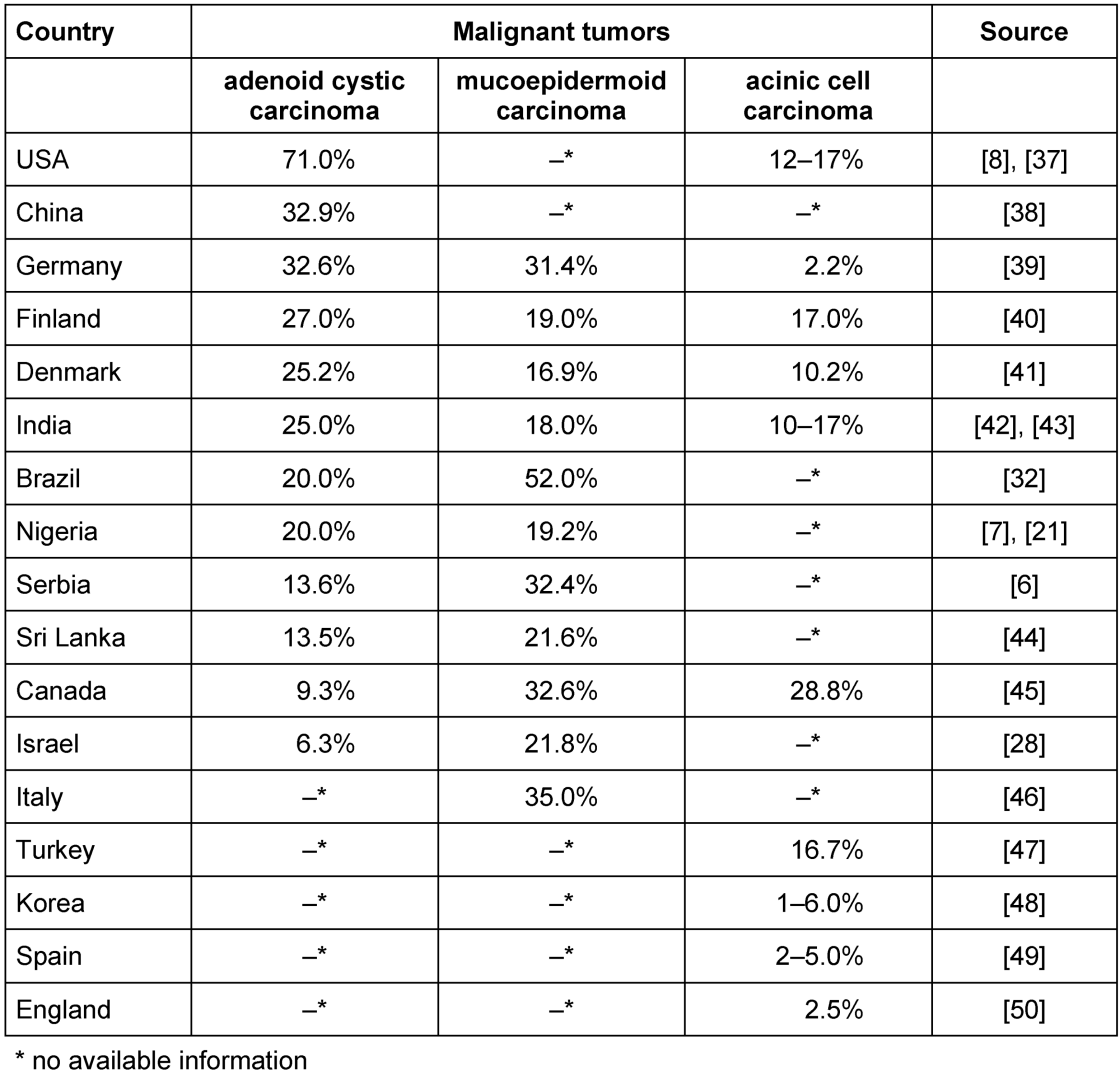
The types of malignant salivary gland tumors – country-specific distribution

**Table 3:**
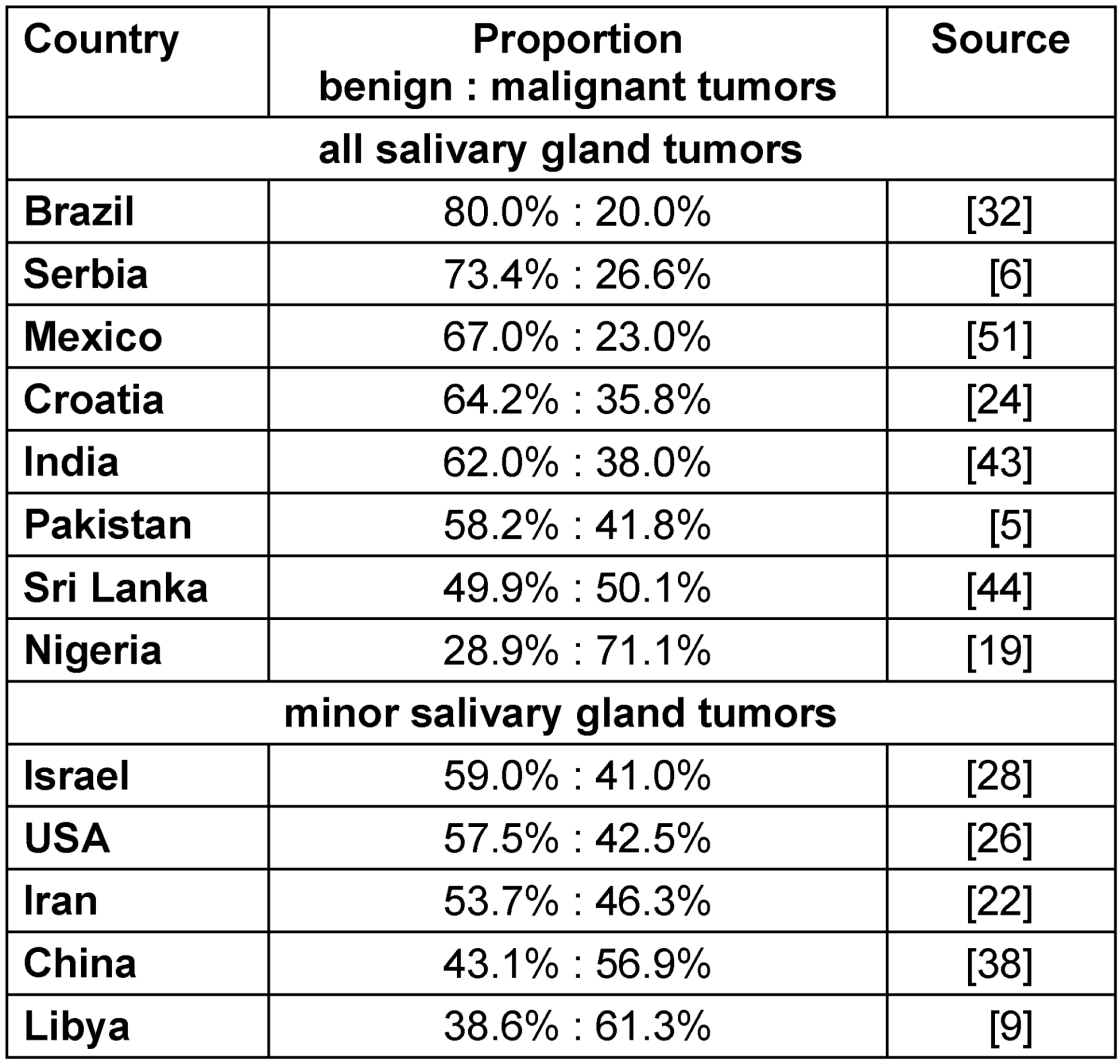
The different occurrences of minor and major salivary gland tumors worldwide

**Table 4:**
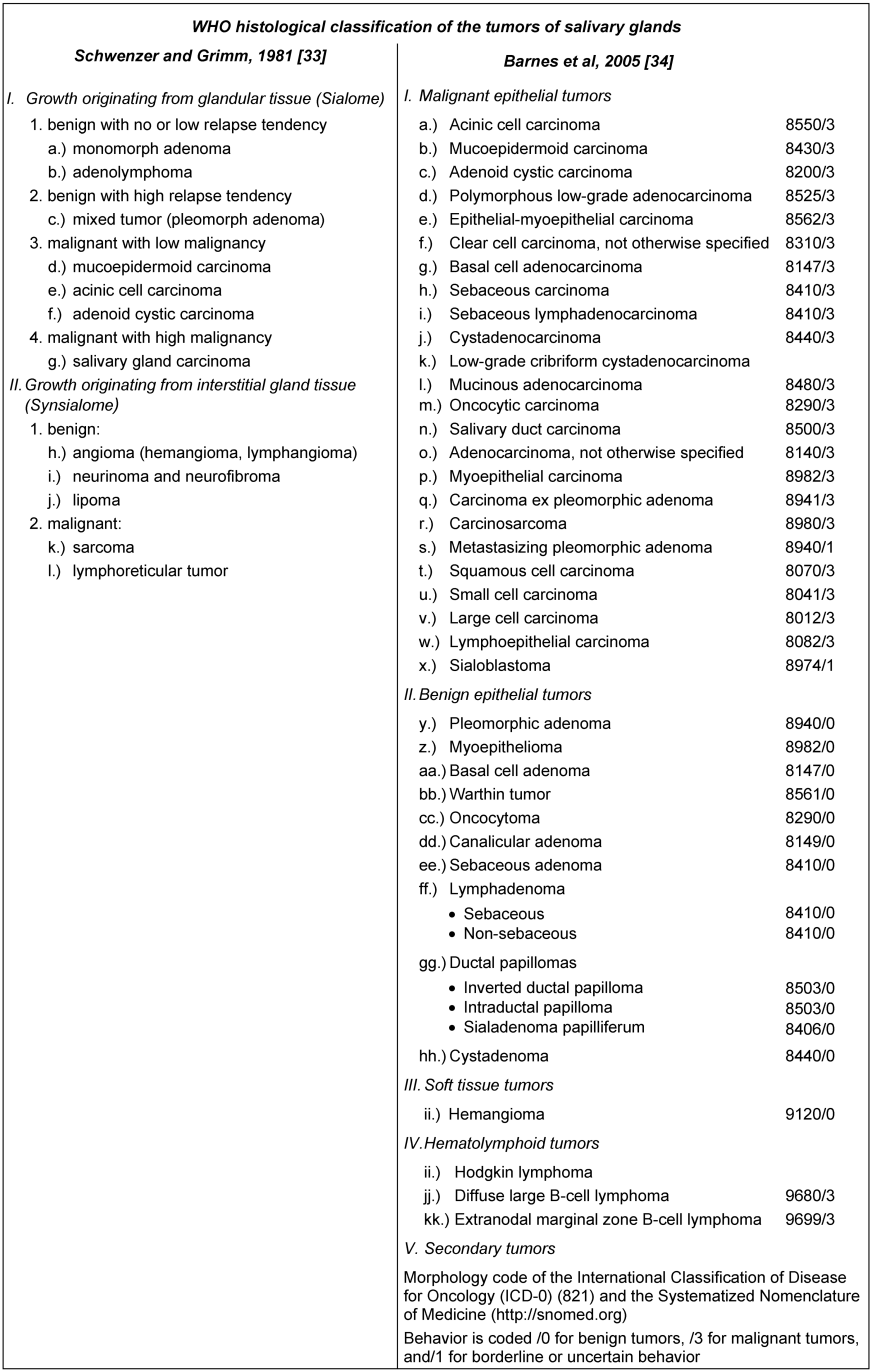
WHO detailed classification of salivary gland tumors (according to Schwenzer/Grimm and Barnes)
